# Mechanistic studies on the role of CHI3L1 in eosinophilic inflammation in chronic sinusitis

**DOI:** 10.3389/fimmu.2025.1562546

**Published:** 2025-03-25

**Authors:** Ling Guo, Yi Peng, Cheng Yang, Xinghong Liu, Weilan Xiong, Weijiang Liao, Jiangang Fan

**Affiliations:** ^1^ Department of Otolaryngology Head and Neck Surgery, Sichuan Provincial People's Hospital, University of Electronic Science and Technology of China, Chengdu, China; ^2^ Department of Otolaryngology Head and Neck Surgery, Chengdu Second People's Hospital, Chengdu, China; ^3^ Department of Otolaryngology Head and Neck Surgery, Sichuan Provincial People's Hospital, Chengdu University of Traditional Chinese Medicine, Chengdu, China

**Keywords:** CHI3L1, chronic sinusitis, eosinophils, inflammatory mechanisms, immunomodulation

## Abstract

More than 10% of adults suffer from chronic rhinosinusitis (CRS), a chronic inflammatory condition that lowers quality of life, reduces productivity, and shortens work hours. Every year, more than 1 million surgeries are performed worldwide as a result of CRS. In recent years, targeted therapy for CRS has become a hotspot of research at home and abroad and has made significant progress, but CRS still has a high recurrence rate. Therefore CRS urgently needs precise targeted therapy. In the pathological process of CRS, the involvement of eosinophils is an important inflammatory mechanism. And excessive aggregation of eosinophils often leads to severe inflammatory responses. Studies have shown that chitinase 3-like protein 1 (CHI3L1) plays a key role in the activation and migration of eosinophils. This review will combine the latest research results to analyse in detail the biological properties of CHI3L1, its expression pattern in CRS, and the possible mechanisms by which it affects eosinophil aggregation by regulating immune responses and inflammatory processes, which will provide insights into the key role of CHI3L1 in the pathological process of CRS and offer a new target for the treatment of CRS.

## Introduction

Chronic sinusitis is a chronic purulent inflammation of the sinuses over time. According to epidemiological studies, 5% ~ 12% of the world’s population suffer from CRS (1–4). Diagnosed by CT or nasal endoscopy, based on symptoms and clinical assessment, with a prevalence ranging from 1.2% to 6.8% (5–8). It is shown that the definitive diagnosis of CRS needs to be diagnosed with definitive tests. Although appropriate pharmacologic or surgical treatments are currently administered to patients with CRS, according to a cross-sectional analysis of patients with CRS, treatment is incomplete in 40 per cent of patients, leading to disease recurrence and affecting the quality of subsequent survival (9). In recent years, darbepoetin, omalizumab, and mepolizumab (10–12) have been used to target IL-4R α, IgE, and IL-5, respectively, in CRS, but the results are still poor. Therefore, precision targeted therapy for CRS is still imminent.

CRS is characterized by persistent inflammation of the nasal mucosa and sinus tissue with at least two symptoms: nasal congestion and/or runny nose with impaired sense of smell or facial pain or pressure lasting at least 12 weeks (4, 13). CRS can be clinically categorized into two types, chronic sinusitis with nasal polyps (CRSwNP) and chronic sinusitis without nasal polyps (CRSsNP) (14). Although the clinical presentation of the two types is very diverse, they frequently display distinct pathological characteristics and inherent pathogenic pathways despite having the same phenotype. Another classification is based on endophenotypes, which are groups characterized by certain disease processes and/or biological markers. Based on the variation in the expression content among the main cytokines in the patient’s endophenotype, we divide CRS into three groups: type 1, which is mediated by the T1 cytokine IFN-γ; type 2, which is mediated by the T2 cytokines IL-5 and eosinophilia; and type 3, which is mediated by the T3 cytokine IL-17A (15, 16). CRS can be further divided into two categories: eosinophilic CRS (ECRS) and non-ECRS (NECRS), depending on the proportion of eosinophils in the nasal mucosal tissue (17). Increased tissue eosinophil levels and a Th2-type inflammatory response, which is frequently linked to allergy illness, are characteristics of ECRS (18). The incidence and prevalence of eosinophilic chronic sinusitis vary widely according to the region and ethnicity. Previous studies have reported that eosinophilic chronic sinusitis is predominantly found in patients with chronic sinusitis in Western countries, accounting for up to 80% of all patients with chronic sinusitis. In Asian countries, such as China and Japan, eosinophilic chronic sinusitis is found in less than 50% of all patients with chronic sinusitis (19). Most of the patients had eosinophilia, and it was recently discovered that the endophenotype of CRSwNP in Asia gradually changed to type 2 inflammation (20, 21). It is hypothesized that the westernization of lifestyles in Asian nations is the cause of this growing tendency. According to Wirach Chitsuthipakorn et al.’s usage of cut-off values and standardized definitions, the percentage of ECRS in Asia may not differ from that in Europe and the US (22). The prevalence of ECRS and the ensuing rise in CRS severity are no longer limited to the West; they are now a global issue. Precision targeted therapy for ECRS is therefore urgent. A growing body of research in recent years has demonstrated the critical role eosinophils play in the pathophysiology of chronic sinusitis. Natural immune granulocytes called eosinophils are important in type 2 inflammation. Numerous active chemicals found in eosinophils can be released from the cell in response to external stimuli, resulting in a range of healthy or pathological reactions. Four granule-associated proteins have been identified in eosinophils, including major basic protein (MBP), eosinophil cationic protein (ECP), eosinophil peroxidase (EPO), and eosinophil-derived neurotoxin (EDN), which have been shown to have the potential to inhibit the growth of bacteria and resist the survival of parasites (23–25). Conversely, these proteins are also cytotoxic, and their excessive release not only elicits an inflammatory response, but also damages local tissues and promotes the formation of nasal polyps (26, 27). In addition, eosinophil activation releases a large number of inflammatory mediators that remodel the vascular epithelium, increase vascular permeability, and allow fluid to leak out, leading to fluid collection in the nasal mucosa further leading to mucosal swelling. In chronic sinusitis with eosinophilia, the eosinophil infiltration environment further leads to the above situation, further aggravating the formation and development of nasal polyps (27). Although the exact cause of ECRS is unknown, it is presently thought that S. aureus and its enterotoxins stimulate the Th2 system in order to encourage the generation of IgE and eosinophil infiltration via a number of different mechanisms (28). The buildup of fibrin webs in nasal polyps results from the simultaneous activation of the coagulation system and inhibition of the fibrinolytic system. The formation of nasal polyps is likely caused by the disruption of the fibrinolytic system, which impedes the fibrinolytic pathway and results in excessive fibrin deposition. This is supported by the fact that the concentration of t-PA in the leptomeningeal tissues of patients with CRSsNP and patients with a simple deviated septum was significantly higher than that in the nasal polyp tissues of patients with CRSwNP. Nasal polyps develop as a result of the excessive fibrin deposition caused by this (29, 30). Therefore, modulation of relevant targets controlling fibrin deposition may be a novel approach for the treatment of ECRS.

CHI3L1, another name for YKL-40, is a glycoprotein implicated in a number of inflammatory conditions and tissue remodeling processes. According to earlier research, patients with atopic dermatitis or asthma have noticeably higher serum levels of CHI3L1, indicating that CHI3L1 is linked to allergic disorders (31, 32). Through its effects on fibrosis, tissue remodeling, and immune cell recruitment, it is also thought to play a role in the pathophysiology of the illness. According to recent research, CHI3L1 is crucial for controlling the fibrinolytic system. In particular, the Th2 inflammatory response is upregulated by CHI3L1 (33). By encouraging macrophage differentiation toward the M2 type, disruption of the Th1/Th2 balance increases fibrin deposition in the ECRS. As a biomarker, elevated levels of CHI3L1 have been linked to eosinophilic respiratory conditions like asthma (31, 34, 35). It is clear that the importance of CHI3L1 in inflammatory diseases cannot be ignored.

This review will delve into the specific mechanism of action of CHI3L1 on eosinophilic inflammation in CRS. We will review the existing research results, analyse the structure, expression and its function of CHI3L1, analyse the role of CHI3L1 in various diseases, especially its expression in CRS and its mechanism of action, and analyse how CHI3L1 affects the pathological process of CRS by regulating the function of eosinophils. In addition, we will discuss the possibility of CHI3L1 as a potential therapeutic target and the direction of future research. Through these discussions, we hope to provide new ideas and strategies for the diagnosis and treatment of CRS.

## Biological properties of CHI3L1

### Structure of CHI3L1

**Figure 1:**
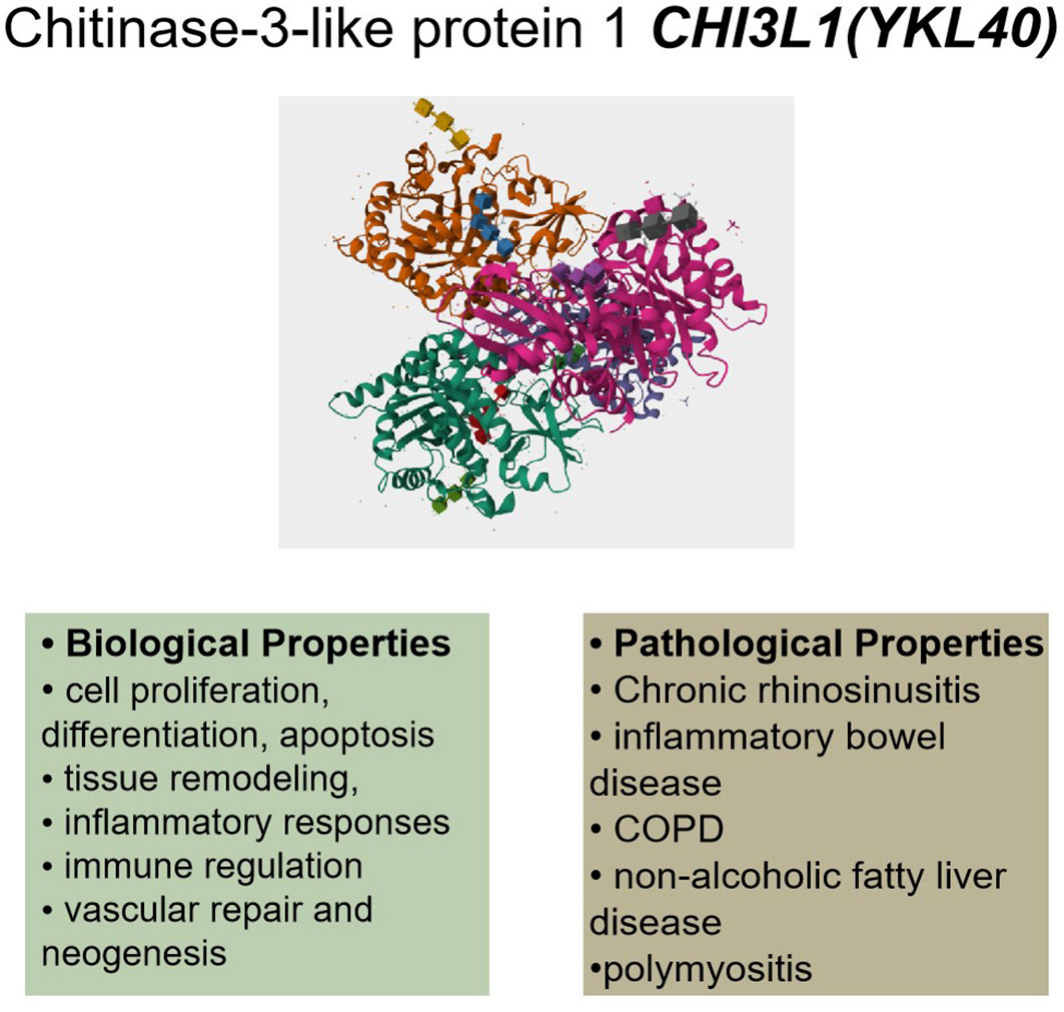
CHI3L1’s (YKL40) biological and pathological characteristics. Cell division, apoptosis, tissue remodeling, inflammatory reactions, immunological control, vascular healing, neogenesis, and cell proliferation are some of its biological characteristics. Pathological diseases such as chronic sinusitis, inflammatory bowel disease, COPD, non-alcoholic fatty liver disease, and polymyositis have been linked to dysregulation of CHI3L1. (This protein structure is from The UniProt Knowledgebase (UniProtKB), Entry:P36222).

A highly conserved glycoprotein that belongs to the family of chitinase 3-like proteins, CHI3L1 is a single polypeptide chain consisting of 383 amino acid residues with an isoelectric point of 7.6 (36, 37). It was first identified in mouse breast cancer cells and is referred to as mammary degradation protein 39 (BRP39) in mice (38). It is a 40 kDa secreted mammalian glycoprotein that can bind chitin but does not have the enzyme activity to catalyze chitin. A chitinase-like structural domain, a C-terminal glycosylation site, and an N-terminal signal peptide are all present in its molecular structure (39). It has recently been determined what CHI3L1’s native structure is. Four separate CHI3L1 monomers that interact with one another in a highly comparable way make up the structure (**Figure 1**). The four monomers contain 117 water molecules and 22–383 residues. One noteworthy feature of the CHI3L1 crystal structure was the lack of a leading sequence from the first 21 N-terminal residues. The ability of CHI3L1 to bind chitin, as previously mentioned, but the absence of enzymatic activity to catalyze chitin is also explained by this structure.The structural domains of barrel glycoside hydrolase family 18 (GH18), which are typical of chitinases and chitinase-like proteins, are also present in the crystal structure of CHI3L1 (40, 41). The conserved cysteine residues seen in the crystal structure of CHI3L1 are also generated via a variety of disulfide bonds. These residues are thought to be a significant factor in the stability and biological function of CHI3L1 and can aid in the crystal structure’s development (42, 43). It is interesting to note that the researchers discovered that two of the three cis-peptide linkages in the crystal structure of CHI3L1 were situated in the sugar-binding motif (44). CHI3L1 is N-glycosylated at Asn60 and has two β(1, 4)-linked Glc NAc residues. After rigorous analysis using the electron densitometry technique, it was discovered that there was a particularly noticeable sequence mismatch at position 311, where an isoleucine residue rather than the predicted threonine was present. The researchers used mass spectrometry to demonstrate that these sequence discrepancies were similar in all four asymmetric CHI3L1 monomers. We have a foundation for understanding the biological role and possible therapeutic benefit of CHI3L1 thanks to the clarification of its crystal structure and the variations in sequence between its four asymmetric monomers.

CHI3L1, a candidate gene for type 2 airway inflammation, may increase eosinophils, leading to a subsequent cascade of inflammation. In recent years, with many advances in genetic studies of CHI3L1 (45–47), CHI3L1 has been found to be associated with susceptibility to chronic sinusitis. CHI3L1 is a candidate gene for type 2 airway inflammation, which leads to an increase in eosinophils and triggers a cascade of inflammation. CHI3L1 gene polymorphisms regulate the expression of CHI3L1 in the airways, and the CHI3L1 protein is encoded by the chitinase 3-like 1 gene CHI3L1. Single-nucleotide polymorphisms (SNPs) in the CHI3L1 promoter are associated with elevated serum levels of CHI3L1 (32, 46). Single nucleotide polymorphisms (SNPs) affecting CHI3L1 levels also influence the progression of chronic sinusitis (48).

### CHI3L1 functions

**Figure 2:**
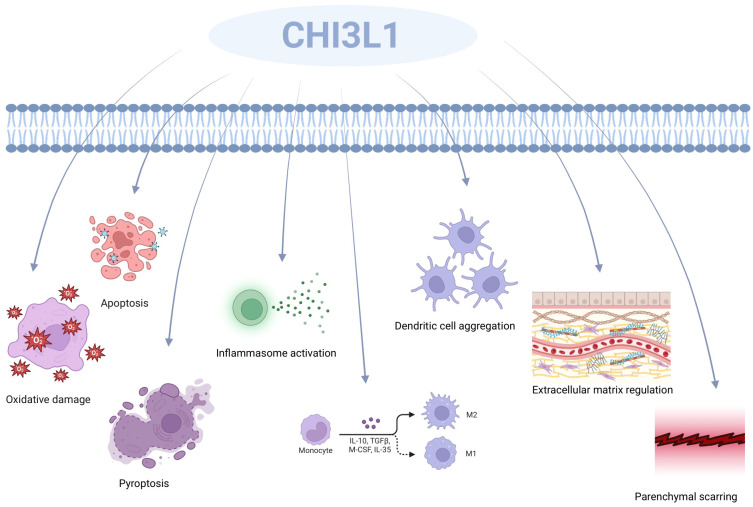
CHI3L1’s function includes oxidative damage, apoptosis, cellular pyrolysis, activation of inflammatory vesicles, development of M2 macrophages, aggregation of dendritic cells (DC), modification of the ECM (ECM), and the production of parenchymal scars.

A comprehensive review of the literature has led to the conclusion that CHI3L1 is produced in a wide variety of cells, such as human synoviocytes (49), osteosarcoma cells (50), fibroblasts (51), endothelial cells (52), chondrocytes (53), smooth muscle cells (36), macrophages (54), neutrophils (55), and colonic epithelial cells (56). It was found that CHI3L1 has very important roles in both physiological and pathological states, which proves that CHI3L1 plays an important role in the study of health and disease (57) (**Figure 2**). CHI3L1 defends against infections and responds to oxidative induction and antigen presentation-induced damage. It coordinates tissue remodeling and repair, controls the release of inflammatory mediators, and plans the migration and aggregation of inflammatory cells. In particular, CHI3L1 regulates basic biological functions such as oxidative damage, apoptosis, cellular pyroptosis, activation of inflammatory vesicles, differentiation of M2 macrophages, aggregation of dendritic cells (DC), modulation of ECM (ECM), and creation of parenchymal scars (39, 58, 59). Additionally, CHI3L1 chemotactically affects smooth muscle and vascular endothelial cells. Through the promotion of vascular smooth muscle cell migration and the stimulation of endothelial tubule formation, its regulatory actions also extend to the regulation of vascular endothelial cell shape (60, 61).

**Figure 3:**
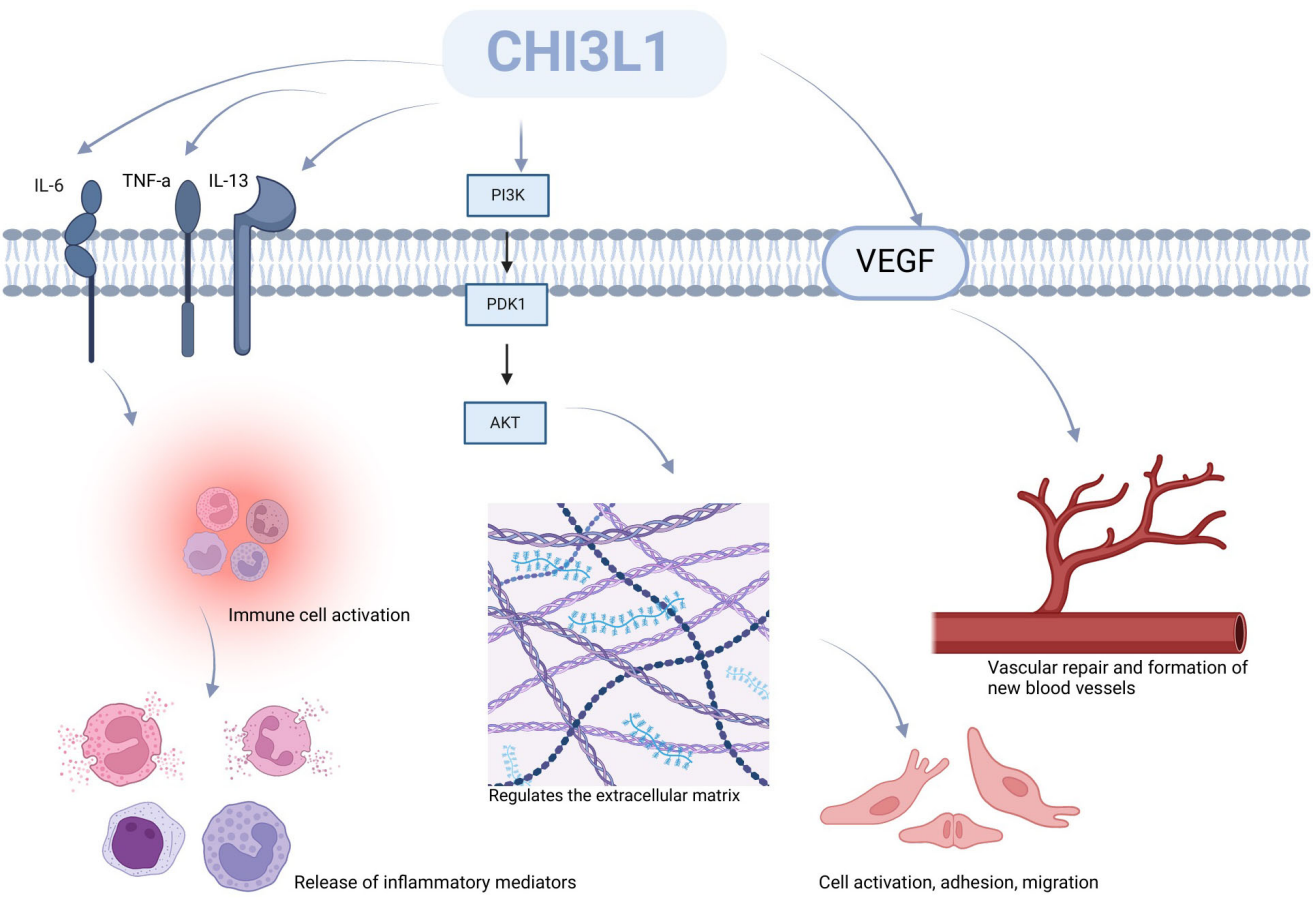
CHI3L1’s function in the inflammatory response. Pro-inflammatory cytokines including interleukin 6 (IL-6), interleukin 13 (IL-13), and tumor necrosis factor-alpha (TNF-alpha) are expressed more when CHI3L1 is present. This causes a number of immune cells, including neutrophils, monocytes, and eosinophils, to become activated.Via PI3K/Akt activation, CHI3L1 influences cell adhesion, migration, and the architecture of ECM proteins like collagen, fibronectin, and proteoglycans among other things. Furthermore, via influencing endothelial cell function and angiogenic signaling pathways, CHI3L1 facilitates vascular healing and neointima development.

CHI3L1 also plays a significant function in the inflammatory response (**Figure 3**). According to research, CHI3L1 may have a role in maintaining a balance between pro- and anti-inflammatory signaling via regulating anti-inflammatory cytokines in physiological settings. Strong external stressors have the ability to enhance the expression of pro-inflammatory cytokines such tumor necrosis factor-α (TNF-α), interleukin 6 (IL-6), and interleukin 13 (IL-13) (62), which can activate numerous immune cells, including neutrophils, monocytes, and eosinophils, via mediating downstream signaling pathways implicated in inflammatory responses. Numerous inflammatory substances are released when these cells are activated by CHI3L1, disrupting the normal cellular barrier and causing a cascade of immunological responses that are dysregulated (59, 63, 64).

In addition to immunomodulation, CHI3L1 has also been implicated in the regulation of ECM components. It was found that CHI3L1, in regulating the extracellular matrix (ECM), may transmit relevant signals through activation of the PI3K/Akt (41, 65) signaling pathway, inducing cells to secrete ECM proteins, including, i.e., collagens, non-collagenous proteins, elastin, proteoglycans, and aminoglycans, and that the cells continue to remodel the ECM through synthesis, degradation, and reorganization mechanisms in development, wound healing, and in certain disease states. The dynamics of the ECM are particularly well characterized (66). Furthermore, CHI3L1 stimulates fibroblast migration, activation, and proliferation inside the organism. The activation, proliferation, and release of active substances like fibrinogen by fibroblasts during CHI3L1 stimulation may stimulate the creation of ECM proteins, hence facilitating tissue remodeling and healing. Additionally, CHI3L1 may regulate the morphology of vascular endothelial cells from vascular repair to angiogenesis by promoting the migration of vascular smooth muscle cells, stimulating the formation of endothelial tubules by endothelial cells, and inducing the production of vascular endothelial growth factor (VEGF) signaling pathway (61). By triggering the particular signaling pathways listed above, CHI3L1 may cause cells to release pro-angiogenic molecules during tissue repair, thus encouraging angiogenesis. High levels of CHI3L1 expression are strongly associated with the severity and course of chronic inflammatory disorders, indicating a critical function for this protein in inflammation regulation.

## Expression pattern of CHI3L1 in chronic sinusitis

### Expression of CHI3L1 in patients with chronic sinusitis

The pathogenesis of CRS is currently the subject of much debate, but most believe (67, 68) that it is due to immune dysfunction, leading to a series of mechanisms that result in the formation of nasal polyps. Although a great deal of research has been conducted on CHI3L1 in immune disorders (69, 70), In particular, Chun Geun et al. (71)in their study verified the establishment of a novel regulatory role of CHI3L1 in the initiation and effector phases of Th2 inflammation and remodeling by *in vitro* modeling the production and characterization of CHI3L1/mice, CHI3L1 transgenic mice, and mice that lacked CHI3L1 and produced CHI3L1 only in the lung epithelium and revealed CHI3L1 to be a Th2 and therapeutic targets for macrophage-mediated diseases, and we know that chronic sinusitis is caused by dysregulated Th2 immunity. In the past, a large number of scholars have investigated the role of CHI3L1 in CRS. Wang et al. simulated immunoregulation in real human ECRS by creating an *in vitro* model of ECRS and verified that CHI3L1 is one of the major genes upregulated in patients with ECRS by single-cell sequencing (38). In addition, in nasal polyp tissue from patients with CRSwNP, CHI3L1 levels were found to be significantly higher than in patients with CRSsNP and with deviated septum alone, and it was revealed that epithelial cells are one of the major cells for CHI3L1 production (72). An *in vitro* model of CRS with eosinophilia was again created, and after treatment with anti-CHI3L1 antibody, it was found that eosinophils in the nasal mucosa of the *in vitro* model were greatly reduced, inflammatory mediators were significantly reduced, and there was a significant reduction in the distribution of cup cells as well as a decrease in mucus secretion, leading to a significant improvement in epithelial cell thickening.Moreover, in nasal polyps treated with anti-CHI3L1 antibody, we found that the expression of TNF-α and eotaxin - 1 was also significantly decreased, suggesting that CHI3L1 may activate signaling pathways through both TNF-α and eotaxin - 1 pathways, inducing eosinophil activation, migration, and release of inflammatory mediators, leading to the development of disease (73). Thus, CHI3L1 is highly associated with eosinophilic chronic sinusitis, and whether CHI3L1 also causes eosinophilia through other mechanisms in patients with chronic sinusitis and nasal polyps needs to be further investigated. Ma et al. isolated and cultured human nasal mucosal epithelial cells, stimulated them with different concentrations of IL-4, and found that CHI3L1 was substantially elevated in patients with chronic sinusitis with polyps, in patients with CRSsNP, and in patients with simple septal deviation, especially in those with CRSwNP. The expression of CHI3L1 was inhibited by other pro-inflammatory cytokines, and lipopolysaccharide (LPS) and dexamethasone caused a significant decrease in the expression of CHI3L1 in human nasal mucosal epithelial cells (72). Therefore, CHI3L1 knockout mice can be used to study the role of CHI3L1 *in vivo*. This will help to elucidate the specific role of CHI3L1 in the pathogenic process involved in ECRSwNP and to design new therapeutic strategies to manage these CRS patients.

### CHI3L1 expression in animal models

In an animal model of chronic sinusitis, the expression pattern of CHI3L1 was consistent with observations in clinical samples. A significant increase in CHI3L1 expression in the nasal mucosa was found by inducing a mouse model of sinusitis. This increase was closely associated with the aggregation of inflammatory cells and the release of inflammatory mediators, further validating the key role of CHI3L1 in the inflammatory response. In addition, reducing CHI3L1 expression by gene knockout or pharmacological intervention significantly reduces inflammatory symptoms, By generating CC10-knockout purebred mice, Wang et al. established a mouse model of ECRS. It turned out that CC10-knockout ECRS mice demonstrated a marked increase in inflammatory cells and inflammatory mediators in sinus tissues in contrast with wild-type mice (38). The expression of CC10 in the nasal mucosa acts through different cytokines that regulate mRNA stability and the expression of certain transcription factors. In addition, CC10 can inhibit the expression and regulation of a variety of cytokines through certain mechanisms and can also inhibit the activity of phospholipase A2, thus attenuating the production and aggregation of inflammatory cells. Lee et al. (74)found by animal modeling that CC10 gene-deficient mice exhibited Th2-type inflammation and IL-13 effector responses by inducing eosinophil chemotaxis and aggregation, increasing IgE production, DC accumulation, and activation, as well as inhibiting inflammatory cell apoptosis and CD95 expression, and inducing PKB/AKT activation due to elevated levels of CHI3L1.It has been found that in human nasal polyps, CC10 gene expression is most significantly down-regulated (75), which leads to an increase in the expression of CHI3L1, inducing eosinophil chemotaxis and the formation of extracellular traps for eosinophils, which cleave and release a large number of cytokines and reactive substances, thus leading to CRS.It is suggested that CHI3L1 may be a potential target for the treatment of chronic sinusitis.

## Role of CHI3L1 in eosinophil recruitment

### Regulation of eosinophil activation and migration by CHI3L1

CHI3L1 (also known as YKL-40) is a glycoprotein secreted by a variety of cell types that plays an important role in CRS. Studies have shown that CHI3L1 expression is significantly increased in ECRS (72), suggesting an important role in eosinophil activation and migration. Eosinophils are key inflammatory cells in CRS, and their overactivation is strongly associated with disease severity and persistence of symptoms. CHI3L1 triggers a series of signalling pathways by binding to receptors on the surface of eosinophils, thereby promoting the activation and functional enhancement of these cells.

In He’s study, TNF-α was found to synergistically induce CHI3L1 mRNA expression with IL-13 or IL-4, and this effect was more pronounced when TNF-α was combined with IL-13 (34). CHI3L1 is able to bind to interleukin-13 receptor a2 (IL-13Ra2) on the surface of eosinophils and activate the PI3K/Akt and MAPK signalling pathways (76).Activation of these signalling pathways leads to enhanced degranulation of eosinophils and release of large amounts of inflammatory mediators such as histamine, leukotrienes and cytokines. This activation is particularly evident in eosinophil-associated diseases such as chronic sinusitis, suggesting a key role for CHI3L1 in disease progression. Further studies have shown that inhibition of CHI3L1 expression significantly reduces the activation status of eosinophils, thereby reducing inflammatory symptoms.

CHI3L1 not only affects the activation of eosinophils, but also significantly regulates their chemotaxis. CHI3L1 affects the expression and function of chemokines, a class of small molecule proteins that regulate cell migration, inflammation and immune response, through a variety of mechanisms.CHI3L1 can regulate chemokine signalling by interacting with other proteins in the cell. For example, CHI3L1 can bind to STAT3 (signal transducer and activator of transcription 3) and affect its phosphorylation and activation status, thereby regulating the expression of chemokine-related genes (77). This intracellular regulatory mechanism allows CHI3L1 to regulate chemokine function at a finer level. It has been shown that CHI3L1 is able to promote the release of chemokines from eosinophils by binding to receptors on their surface, such as CD44 and RAGE, thereby enhancing the migration of eosinophils towards inflammatory regions (65). This chemotaxis is particularly evident in eosinophil-associated diseases such as chronic sinusitis, suggesting a key role for CHI3L1 in disease progression. Further studies have shown that inhibition of CHI3L1 expression significantly reduces eosinophil chemotaxis, thereby attenuating inflammatory symptoms.

## Potential application of CHI3L1 in the management of chronic sinusitis

### Drug development targeting CHI3L1

In recent years, targeted drugs against CHI3L1 have made significant progress in the treatment of various diseases (**Table 1**). Studies have shown significant achievements in targeted therapy of recurrent brain tumors (78), breast cancer (79), airway inflammatory diseases (80), and glioblastoma (81).

**Table 1:** Drug development targeting CHI3L1.

Veterinary drug	Target point	Corresponds	Bibliography
The mTOR inhibitor rapamycin and the STAT3 inhibitor	Targeting CHI3L1 - STAT3 - mTOR	Inhibition of glioma growth in TMZ-R recurrence	(78)
Neutralising anti-CHI3L1 antibody	Targeting the KR-rich structural domain	Inhibition of angiogenesis and tumour cell migration in breast cancer	(79)
Anti-CHI3L1 antibody	Neutralisation CHI3L1	Reduction of IL-13-dominated airway inflammation during RSV infection	(80)
Anti-CHI3L1 antibody	VEGF receptor 2	Glioblastoma Tumor Growth Inhibition	(81)

Based on the critical role of CHI3L1 in CRS, drug development targeting CHI3L1 has become a research direction of great interest. Several strategies have been proposed to inhibit the activity of CHI3L1, including monoclonal antibodies, small molecule inhibitors, and gene therapy. Monoclonal antibodies act directly on the CHI3L1 protein to inhibit the inflammatory response by blocking its binding to the receptor. Small molecule inhibitors, on the other hand, achieve therapeutic effects by interfering with the synthesis or secretion of CHI3L1. Gene therapy, on the other hand, aims to radically reduce the role of CHI3L1 in the inflammatory process by modulating its expression level.

Although the development of drugs targeting CHI3L1 is still in the early stages, initial preclinical studies have shown some potential. For example, certain monoclonal antibodies significantly reduced the inflammatory response of the sinus mucosa and improved associated symptoms in animal models. However, the safety and efficacy of these drugs still need to be validated in further clinical trials. In addition, how to choose the appropriate route and dose of administration and how to evaluate the therapeutic effect are also issues that need to be addressed in future studies. Overall, drug development targeting CHI3L1 offers new hope for the treatment of chronic sinusitis. With further research, it is expected that safer and more effective therapeutic regimens will be developed in the future, thus improving the clinical prognosis and quality of life of patients.

### The potential of CHI3L1 as a biomarker

CHI3L1 is a protein associated with a variety of diseases, and its use as a biomarker has gradually increased in recent years (**Table 2**). CHI3L1 expression levels are significantly elevated in a variety of inflammatory diseases, tumors, and neurodegenerative disorders, making it a potential biomarker. Examples include polymyositis/dermatomyositis-associated interstitial lung disease (82), non-alcoholic fatty liver disease (NAFLD) (83), inflammatory bowel disease (IBD) in children (84) and adults (85), endothelial dysfunction and hypertension in patients with obstructive sleep apnea (86), and diabetes (87).

**Table 2:** The potential of CHI3L1 as a biomarker.

Systems	Diseases	Affect
Respiratory, immunization	Interstitial lung disease associated with polymyositis/dermatomyositis	CHI3L1↑ (82)→lung function↓
digestion	Nonalcoholic Fatty Liver Disease	CHI3L1↑ (83)→liver function↓
digestion	inflammatory bowel disease	CHI3L1↑ (84, 85)→ digestion function↓
respiratory	obstructive sleep apnea	CHI3L1↑ (86, 88)→lung function↓
endocrine	diabetes	CHI3L1↑→ (87) hypoglycemia↑

And in the study of CRS, CHI3L1 has also been considered as a possible potential biomarker. CRS is a common upper respiratory disease characterised by chronic inflammation of the sinus mucosa, often accompanied by nasal congestion and purulent discharge. Due to its complex pathological process involving the interaction of multiple immune cells and inflammatory mediators, the search for effective biomarkers is important for the diagnosis and treatment of the disease. Studies have shown that CHI3L1 expression is significantly elevated in nasal secretions and tissues of patients with chronic sinusitis. This high expression was closely related to the severity of the disease and the degree of inflammatory activity.CHI3L1 is not only involved in the regulation of inflammatory responses, but may also affect the structure and function of sinus mucosa by promoting fibrosis and tissue remodelling. In addition, high CHI3L1 expression was associated with eosinophil infiltration, which is an important pathological feature in chronic sinusitis.

Therefore, CHI3L1 not only plays an important role in the pathological process of CRS, but may also be used as a potential biomarker for early diagnosis of the disease, assessment of the condition and monitoring of treatment effects. Future studies should further validate the expression differences of CHI3L1 in different types of CRS and explore its feasibility and accuracy in clinical applications.

## Conclusion

This review methodically investigated the various ways that CHI3L1 contributes to eosinophilic inflammation in CRS, highlighting its crucial functions in immune response mediation, eosinophil function regulation, and possible therapeutic uses. In addition to expanding our knowledge of CHI3L1’s role in the pathophysiology of CRS, these investigations offer a crucial theoretical foundation for upcoming clinical research and treatment approaches. Advances in the study of CHI3L1 represent the in-depth analysis of the illness mechanism by modern medicine at the molecular level. There are still some differences and disagreements in the findings from various studies, despite the fact that a significant number of investigations have been carried out to demonstrate the critical role of CHI3L1 in CRS. For instance, some research highlight CHI3L1’s pro-inflammatory function, while others suggest that it may have anti-inflammatory properties. The variety of experimental designs, sample sources, and disease stages may be the cause of these discrepancies. Therefore, using bigger sample numbers and multicenter cooperation, future research must further validate and improve the mechanism of action of CHI3L1. The evolution of treatment approaches connected to CHI3L1 will be influenced by how these various research viewpoints are balanced and integrated. For instance, CHI3L1 expression can be more accurately controlled by combining gene editing technology with bioinformatics analysis, allowing for more individualized therapy of chronic sinusitis. Furthermore, as CHI3L1 is extensively expressed in many inflammatory disorders, its discovery in chronic sinusitis may also lead to novel treatment approaches for other associated conditions. Thus, research on CHI3L1 offers a wide range of opportunities for interdisciplinary collaboration and the creation of novel treatment approaches in addition to its significant clinical relevance.
